# Dysregulated myosin in Hermansky-Pudlak syndrome lung fibroblasts is associated with increased cell motility

**DOI:** 10.1186/s12931-022-02083-w

**Published:** 2022-06-23

**Authors:** Jewel Imani, Steven P. M. Bodine, Anthony M. Lamattina, Diane D. Ma, Shikshya Shrestha, Dawn M. Maynard, Kevin Bishop, Arinze Nwokeji, May Christine V. Malicdan, Lauren C. Testa, Raman Sood, Benjamin Stump, Ivan O. Rosas, Mark A. Perrella, Robert Handin, Lisa R. Young, Bernadette R. Gochuico, Souheil El-Chemaly

**Affiliations:** 1 Division of Pulmonary and Critical Care Medicine, Brigham and Women’s Hospital, Harvard Medical School, 75 Francis Street, Boston, MA 02115 USA; 2 Medical Genetics Branch, NHGRI, NIH, Bethesda, MD 20892 USA; 3 Division of Hematology, Brigham and Women’s Hospital, Harvard Medical School, Boston, MA 02115 USA; 4 Zebrafish Core Facility, NHGRI, NIH, Bethesda, MD 20892 USA; 5 NIH Undiagnosed Diseases Program, Common Fund, Office of the Director, NIH, Bethesda, MD 20892 USA; 6 Department of Pediatric Newborn Medicine, Brigham and Women’s Hospital, Harvard Medical School, Boston, MA 02115 USA; 7 Division of Pulmonary and Sleep Medicine, The Children’s Hospital of Philadelphia, Perlman School of Medicine at the University of Pennsylvania, Philadelphia, PA 19104 USA

**Keywords:** Hermansky-Pudlak syndrome, Pulmonary fibrosis, Myosin IIB, Cell migration

## Abstract

Hermansky-Pudlak syndrome (HPS) is an autosomal recessive disorder characterized by improper biogenesis of lysosome-related organelles (LROs). Lung fibrosis is the leading cause of death among adults with HPS-1 and HPS-4 genetic types, which are associated with defects in the biogenesis of lysosome-related organelles complex-3 (BLOC-3), a guanine exchange factor (GEF) for a small GTPase, Rab32. LROs are not ubiquitously present in all cell types, and specific cells utilize LROs to accomplish dedicated functions. Fibroblasts are not known to contain LROs, and the function of BLOC-3 in fibroblasts is unclear. Here, we report that lung fibroblasts isolated from patients with HPS-1 have increased migration capacity. Silencing HPS-1 in normal lung fibroblasts similarly leads to increased migration. We also show that the increased migration is driven by elevated levels of Myosin IIB. Silencing *HPS1* or *RAB32* in normal lung fibroblasts leads to increased MYOSIN IIB levels. MYOSIN IIB is downstream of p38-MAPK, which is a known target of angiotensin receptor signaling. Treatment with losartan, an angiotensin receptor inhibitor, decreases MYOSIN IIB levels and impedes HPS lung fibroblast migration in vitro. Furthermore, pharmacologic inhibition of angiotensin receptor with losartan seemed to decrease migration of HPS lung fibroblasts in vivo in a zebrafish xenotransplantation model. Taken together, we demonstrate that BLOC-3 plays an important role in MYOSIN IIB regulation within lung fibroblasts and contributes to fibroblast migration.

**Supplementary Information:**

The online version contains supplementary material available at 10.1186/s12931-022-02083-w.

## Background

Hermansky-Pudlak syndrome (HPS) is a group of rare autosomal recessive disorders in which the formation of lysosome-related organelles (LROs) is defective [1]. LROs are membrane-bound organelles with specific functions in specialized cells that share several features with conventional lysosomes [2]. Genetically, there are eleven distinct types of HPS (HPS1-11), all of which exhibit oculocutaneous albinism and bleeding diathesis of varying severity due to dysfunctional biogenesis of melanosomes and platelet dense granules, respectively [3–5]. HPS-1, HPS-4, and, to a lesser extent, HPS-2 patients are at risk for pulmonary fibrosis, which is the predominant cause of mortality among this population [6–10]. Despite two clinical trials [11, 12], there is no established medical treatment for HPS pulmonary fibrosis, and lung transplantation remains the only viable option [13, 14]. As such, further investigation into the pathogenesis of HPS pulmonary fibrosis is imperative for the discovery of novel therapeutic or prophylactic avenues.

HPS1 and HPS4 proteins assemble to form a heterodimer known as biogenesis of lysosome-related organelles complex-3 (BLOC-3) [1, 15]. BLOC-3 serves as a guanine nucleotide exchange factor (GEF) for Rab32/38 and induces inactive Rab proteins to release GDP and bind to GTP [16]. Previous findings have revealed that deficiency in BLOC-3, Rab32, or Rab38 in lung epithelial cells leads to abnormal membrane protein trafficking and ultimately aggravates bleomycin-induced cell injury [17]. Fibroblasts are the primary effector cells in fibrotic disorders [18, 19], and they contribute to the development of HPS pulmonary fibrosis [20]. However, lung fibroblasts are not known to harbor LROs, and the function of BLOC-3 in lung fibroblasts is not clearly defined.

In the present study, we evaluated the pro-fibrotic phenotypes of HPS lung fibroblasts and identified a Myosin IIB-dependent pathway leading to enhanced HPS lung fibroblast migration. Myosin IIB is known to be upregulated by the activation of the angiotensin II receptor type I (AGTR1) [21]. Blocking the AGTR leads to decreased Myosin IIB levels and impaired migration. Collectively, we found important functional consequences for BLOC-3 defects in human lung fibroblasts, which are manifested by the accumulation of Myosin IIB protein and enhanced pro-fibrotic phenotype, suggesting that AGTR1 could be a promising therapeutic target for the treatment of HPS-1 pulmonary fibrosis.

## Results

### Enhanced HPSLF migration in vitro

**Fig. 1:**
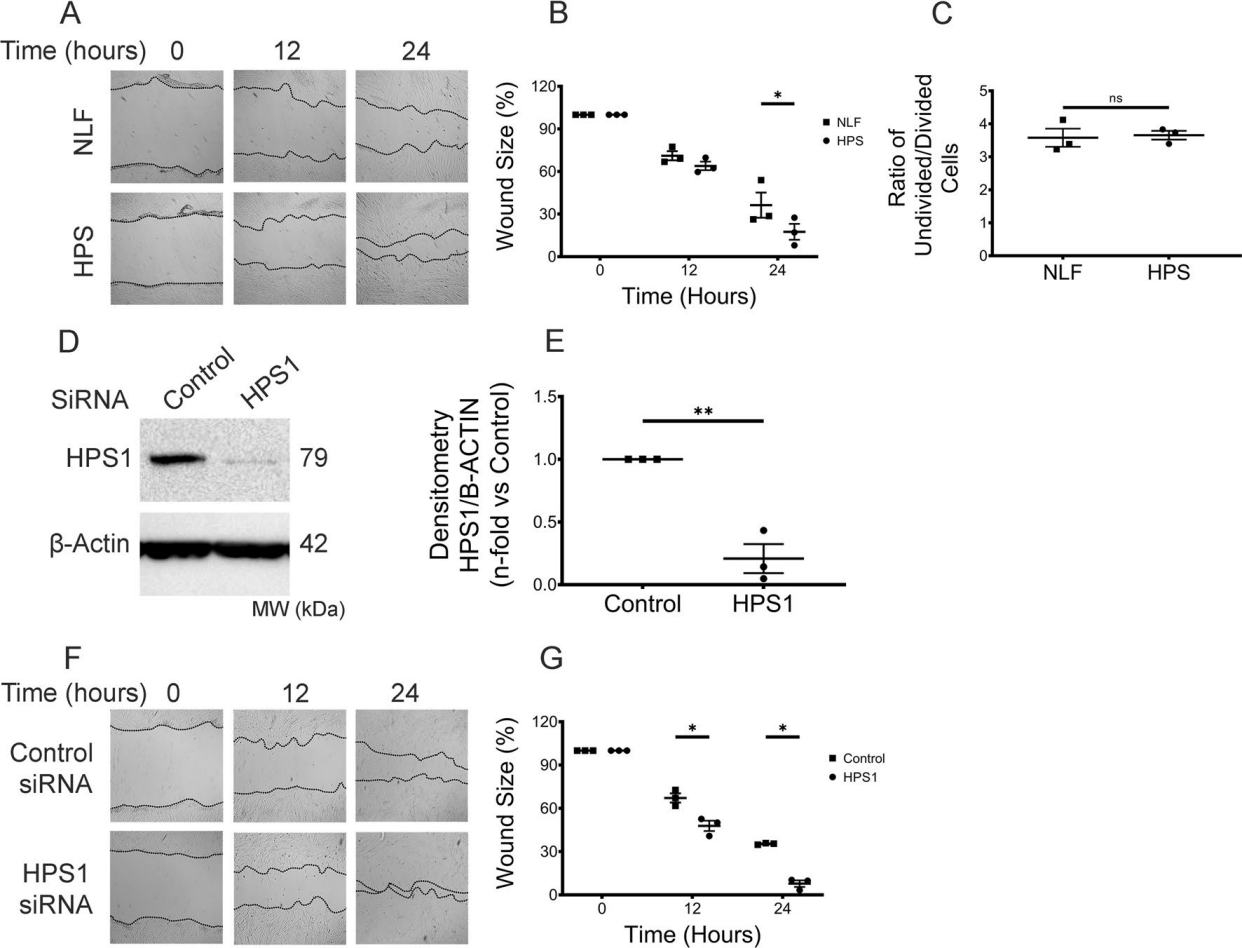
Enhanced HPSLF migration in vitro is HPS1-dependent. **A**, **F** Representative microscopy images of scratch assays at 0, 12, and 24 h after gaps were generated. Scratch assays were performed with **A** NLF (n = 3 technical replicates) and HPSLF (n = 3 technical replicates); **F** NLF transfected with control or HPS1 siRNA; **B**, **G** Quantitative analysis of the migration potential of **B** NLF and HPSLF; **G** NLF transfected with control or HPS1 siRNA. The rate of fibroblast migration was determined by calculating the percentage of open wound area at the indicated time points to that of the corresponding initial scratch. **C** Ratio of CFSE MFI from proliferating cells over undivided cells **D** Western blot analysis of HPS1 protein expression in NLF transfected with either control or HPS1 siRNA. β-Actin was used as a loading control. **E** Ratio of HPS1 to β-Actin density expressed as fold change relative to NLF transfected with control siRNA. Data are expressed as mean ± SEM of three independent experiments. **B**, **G** Data analyzed using two-way mixed ANOVA *P < 0.05. **C**, **E** Data was analyzed using Student’s *t*-test *P < 0.05, **P < 0.01

Given the high prevalence and early onset of pulmonary fibrosis in patients with HPS-1, we examined the pro-fibrotic phenotypes of human HPS lung fibroblasts (HPSLF). We first examined whether the migratory capability of HPSLF was altered using in vitro scratch assays. The rate of gap closure was monitored for 24 h after disruption of the confluent cell monolayer. Within 24 h, areas of denuded surface in HPSLF were considerably smaller compared to that in normal lung fibroblast (NLF), suggesting that cell motility is enhanced in HPSLF (Fig. 1A, B). The efficiency of wound healing in scratch assays can be influenced by both cell migration and proliferation [22]. To take both factors into account, we used flow cytometry to assess cell proliferation by measuring the dilution of CFSE stain over 3 days of cell growth; we found that HPSLF did not possess a proliferative advantage over NLF (Fig. 1C), indicating that the accelerated migration of HPSLF is primarily due to increased cell motility.

Next, to complement our findings in HPSLF, we repeated scratch assays with NLF in which *HPS1* was silenced with siRNA (Fig. 1D, E). Quantification of the residual wound areas revealed that NLF transfected with *HPS1* siRNA acquired augmented migratory potential reminiscent of HPSLF (Fig. 1F, G), thus confirming the impact of HPS1 on fibroblast phenotype.

### Enhanced migration of HPSLF is myosin IIB dependent

**Fig. 2:**
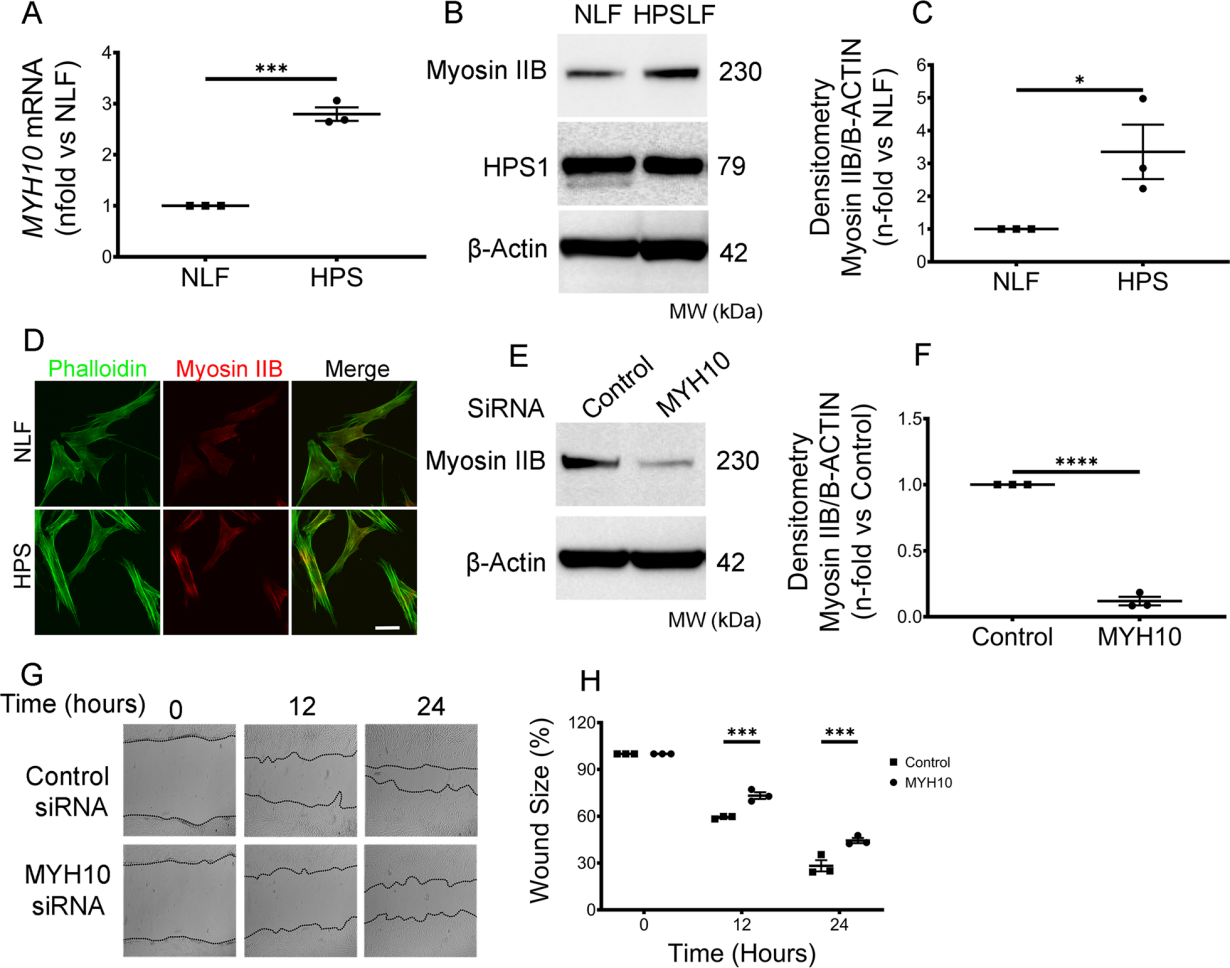
Enhanced migration of HPSLF is Myosin IIB dependent. **A** Real-time PCR analysis of *MYH10* mRNA in NLF (n = 3 technical replicates) and HPSLF (n = 3 technical replicates). Results were expressed as fold change relative to NLF. **B** Western blot analysis of Myosin IIB and HPS1. Protein expression in control NLF and HPSLF cells are shown; β-Actin was used as a loading control. **C** Ratio of Myosin IIB to β-Actin density expressed as fold-change relative to control NLF. **D** Immunofluorescent images of NLF and HPSLF stained with Phalloidin (green) and Myosin IIB (red). **E** Western blot analysis of Myosin IIB protein expressions in HPSLF transfected with either control or *MYH10* siRNA. β-Actin was used as a loading control. **F** Ratio of Myosin IIB to β-Actin density expressed as fold-change relative to control siRNA. **G** Representative microscopy images of scratch assays at 0, 12, and 24 h after gaps were generated in control or *MYH10* siRNA transfected HPSLF. **H** Quantitative analysis of the migration potential of control or *MYH10* siRNA transfected HPSLF. The rate of fibroblast migration was determined by calculating the percentage of open wound area at the indicated time points to that of the corresponding initial scratch. Data are expressed as mean ± SEM of three independent experiments. **A**, **B**, **E** Data were analyzed using students *t*-test *P < 0.5, ***P < 0.001, ****P < 0.0001. **G** Data were analyzed using two-way mixed ANOVA ***P < 0.001

Based on these observations, we hypothesized that Myosin IIB, a key motor protein that propels fibroblast migration [23], was responsible for the increased motility of HPSLF. As an initial assessment of Myosin IIB’s involvement in the HPSLF migratory phenotype, we first quantified the expression of *MYH10*, which encodes Myosin IIB. Real-time PCR analysis revealed that *MYH10* gene expression in HPSLF was approximately three-fold higher compared to that of NLF (Fig. 2A). Furthermore, this increase in *MYH10* mRNA corresponded to elevated Myosin IIB protein staining in both western blot and Immunofluorescence microscopy images (Fig. 2B–D). Real-time PCR analysis also revealed that HPSLF have increased gene expression of ECM genes; Elastin, Laminin, and collagens 1 and 3. To further support our results, we also investigated Myosin IIB expression levels in fibroblast cells derived from a second normal and HPS1 patient; we observed a similar elevated expression of myosin IIB protein levels in HPSLF compared to NLF (Additional file 1: Fig. S1 and Additional file 2: Fig. S2A, B).

Since Myosin IIB inhibition has been reported to reduce the motility of numerous other cell types [24–26], we queried whether suppression of *MYH10* expression could impede HPSLF migration. To this end, HPSLF were transfected with siRNA directed against *MYH10* (Fig. 2D, E) and then subjected to scratch assays with images captured at 0, 12, and 24 h after the wounds were inflicted. As anticipated, a side-by-side comparison demonstrated that closure of the scratch wound required significantly more time in Myosin IIB-depleted HPSLF compared to the cells transfected with control siRNA (Fig. 2F, G). Overall, our results support a role for dysregulated Myosin IIB in enhanced migration of HPSLF.

### A BLOC3-dependent mechanism modulates myosin IIB level in HPSLF

**Fig. 3:**
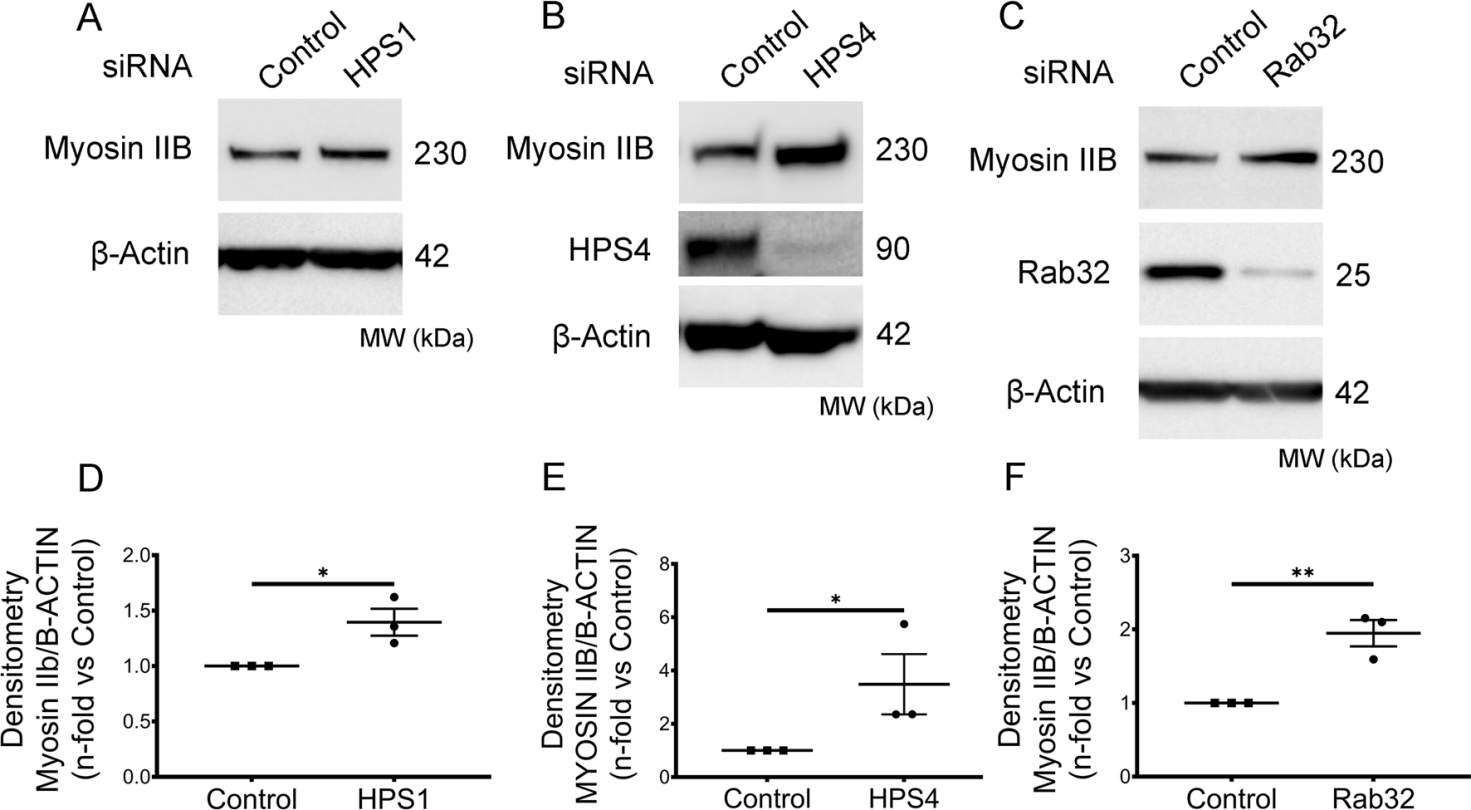
A BLOC3-dependent mechanism modulates Myosin IIB level in HPSLF. **A** and **D** Western blot analysis of Myosin IIB protein expression in NLF (n = 3 technical replicates) transfected with either control or HPS1 siRNA. β-Actin was used as a loading control (same membranes as in Fig. 1D). Ratio of Myosin IIB to β-Actin density expressed as fold change relative to NLF transfected with control siRNA. **B** and **E** Western blot analysis of Myosin IIB protein expression in NLF (n = 3 technical replicates) transfected with either control or HPS4 siRNA. β-Actin was used as a loading control. Ratio of Myosin IIB to β-Actin density expressed as fold change relative to NLF transfected with control siRNA. **C** and **F** Western blot analysis of Myosin IIB in NLF (n = 3 technical replicates) transfected with either control or Rab32 siRNA. β-Actin was used as a loading control. The ratio of Myosin IIB to β-Actin density expressed as fold change relative to NLF transfected with control siRNA. Data are expressed as mean ± SEM of three independent experiments. Data were analyzed using a student’s *t*-test *P < 0.05, **P < 0.01, ****P < 0.0001

HPS1 and HPS4 are two components of BLOC-3, an indispensable guanine nucleotide exchange factor (GEF) for small GTPases Rab32 and Rab38 [16]. To investigate whether BLOC3 proteins could account for the alteration in Myosin IIB levels, we silenced *HPS1, HPS4,* and *RAB32* via siRNA in NLF and validated target depletion by immunoblotting (Fig. 3). Silencing of either component of BLOC-3 or Rab32 resulted in elevated levels of Myosin IIB protein levels. We observed a similar rise in Myosin IIb protein levels in NLF following the use of a different HPS1 siRNA construct (Additional file 2: Fig. S2C, D).

### Pharmacological blockade of MYH10 expression blocks HPSLF migration in vitro

**Fig. 4:**
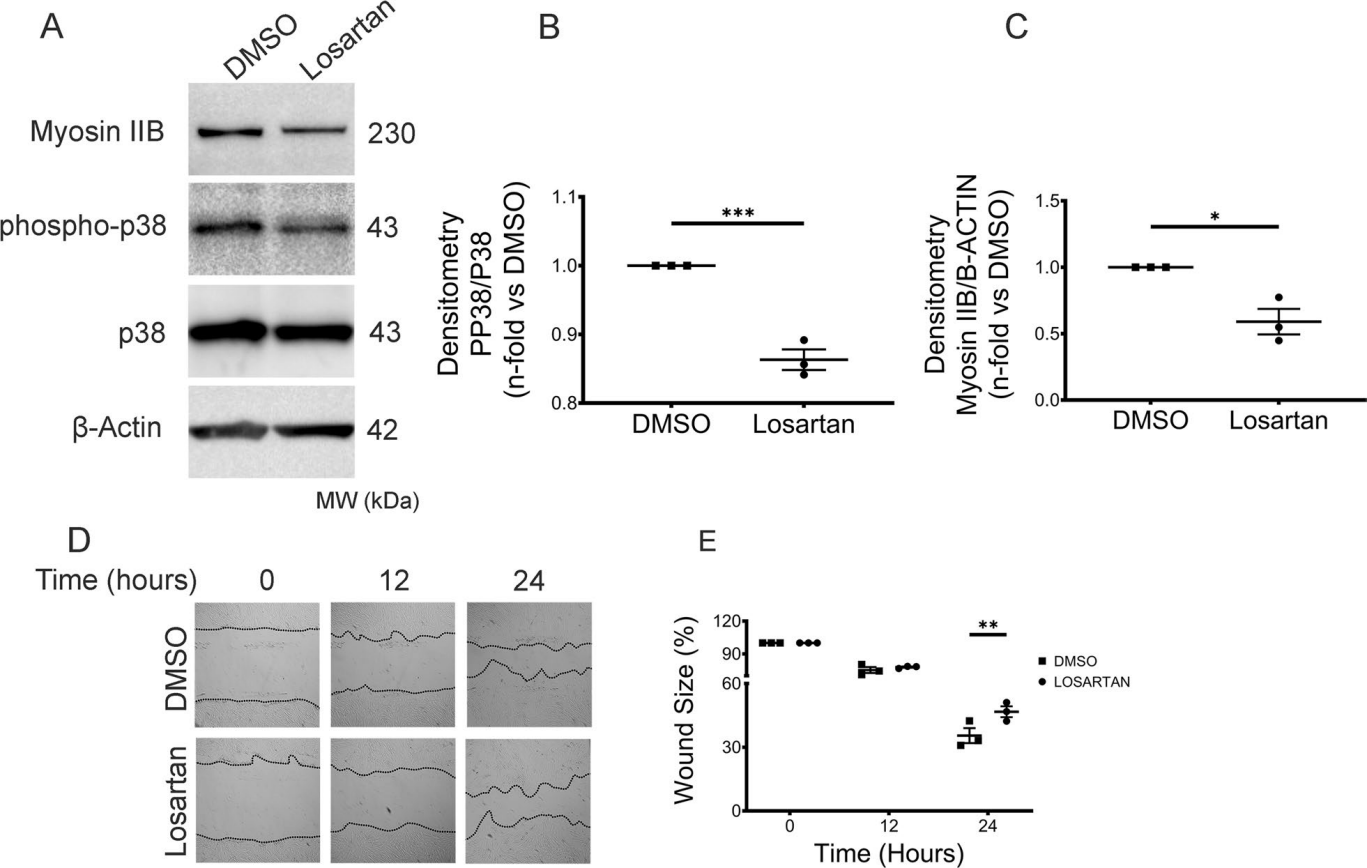
Losartan blocks the enhanced HPSLF migration in vitro. **A** Western blot analysis of Myosin IIB, phospho-p38, and p38 protein expression in HPSLF (n = 3 technical replicates) treated with vehicle (DMSO) or with losartan (100 nM). β-Actin was used as a loading control. **B** Ratio of phospho-p38 to p38 density and **C** Myosin IIB to β-Actin density expressed as fold-change relative to DMSO. **D** Representative microscopy images of scratch assays at 0, 12, and 24 h after gaps were generated. Scratch assays were performed with HPSLF (n = 3 technical replicates) treated with vehicle (DMSO) or losartan (100 nM). **E** Quantitative analysis of the migration potential. The rate of fibroblast migration was determined by calculating the percentage of open wound area at the indicated time points to that of the corresponding initial scratch. Data are expressed as mean ± SEM of three independent experiments. **B**, **C** Data analyzed using students *t*-test *P < 0.5, ***P < 0.001. **E** Data analyzed using two-way mixed ANOVA **P < 0.01

To further explore the link between Myosin IIB and HPSLF migratory phenotype, we explored the regulatory pathways controlling Myosin IIB expression and activity. The p38 mitogen-activated protein kinase (MAPK) pathway is essential in Myosin IIB regulation [27], and p38 is a downstream effector of AGTR1-mediated signaling [28, 29]. Furthermore, Myosin IIB is upregulated by AGTR1-mediated signaling, and *MYH10* expression can be inhibited with common AT1 receptor antagonists such as losartan [21]. To elucidate the relationship between AGTR1 signaling and HPSLF phenotype, we examined p38 phosphorylation in HPSLF incubated with and without losartan. Treatment with losartan [30] significantly abrogated p38 phosphorylation in HPSLF compared to HPSLF control cells treated with vehicle-only (Fig. 4A–C). Myosin IIB expression in these same cells was also significantly reduced in HPSLF treated with losartan compared to control cells. Treating HPSLF with the p38 MAPK inhibitor SB202190 [31] (Additional file 3: Fig. S3A, B) similarly reduced Myosin IIB protein levels. In further support of these results, we also silenced HPS1 and HPS4 in NLF cells (Additional file 3: Fig. S3C–F) and observed increased levels of phosphorylated p38 protein; linking the HPS1 defect to changes in p38 signaling.

To address the potential functional significance of these findings, we measured cell migration of HPSLF incubated with losartan or vehicle-only in scratch assays [30]. We found that cell migration of HPSLF treated with losartan was significantly reduced compared to control HPSLF incubated with vehicle only (Fig. 4D, E). These results demonstrate that pharmacological blockade of Myosin IIB is associated with decreased HPSLF migration.

### Losartan alters HPSLF migration in vivo

**Fig. 5:**
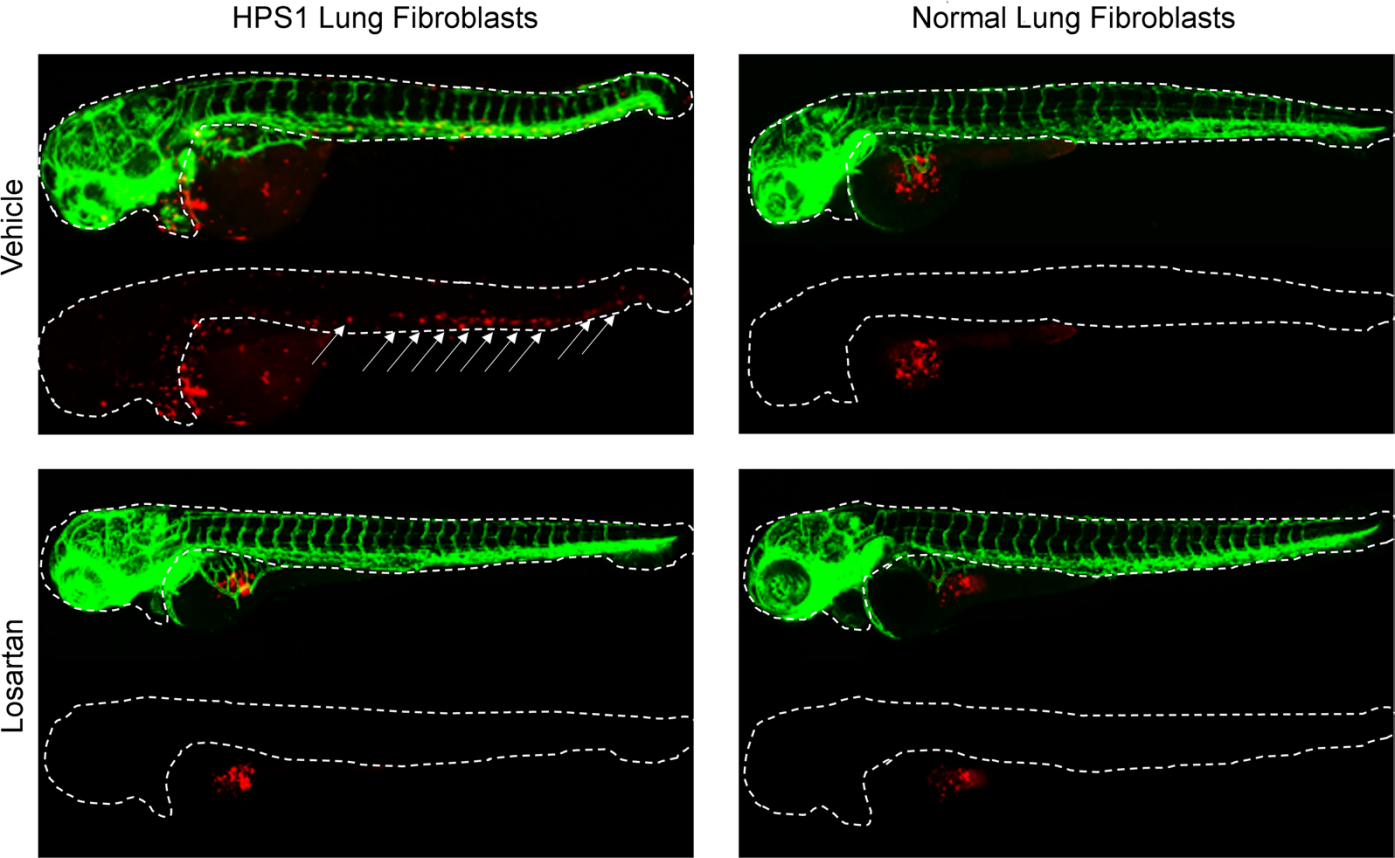
Modulation of enhanced migratory capacity of HPS lung fibroblasts (HPSLF) by losartan in vivo. HPSLF and normal lung fibroblasts (NLF) were treated with losartan or vehicle (PBS) and labeled with a CM-Dil live-cell marker (red). 75 cells were injected into the yolk sac of 48 h post-fertilization Tg(fli1:GFP, green) zebrafish embryos expressing GFP in their blood vessels. HPSLFs exhibit stronger migratory capacity than NLFs at 24 h (not shown) and 48 h after injection. Arrows indicate the migration of labeled fibroblasts from the yolk sac into outlined regions of zebrafish embryos. Treatment with losartan reduces HPSLF migratory capacity compared to baseline

To examine the migration of human lung fibroblasts in vivo, we developed a xenotransplant model, where NLF and HPSLF labeled with fluorescent dye were injected into the yolk sac of zebrafish embryos. Zebrafish embryos were imaged at 24 and 48 h after injection to measure the migration of cells from the yolk sac. Consistent with our in vitro data, HPSLF exhibited increased migration in vivo compared to NLF. Notably, the increased cell migration in zebrafish embryos seemed to be inhibited when HPSLF were pre-incubated with losartan prior to injection (Fig. 5, Additional file 4: Fig. S4).

## Discussion

In this study, we demonstrate for the first time that deficiency in HPS1, a subunit of BLOC-3, results in Myosin IIB protein expression in HPSLF. We also demonstrate that elevated levels of Myosin IIB in HPSLF is associated with increased cell motility and that blockade of AGTR1 using losartan corrects the pro-fibrotic phenotype. Of significant clinical interest, losartan is an FDA-approved AGTR1 antagonist for the treatment of hypertension, left ventricular hypertrophy, and diabetic nephropathy, and it has been clinically used for more than two decades with an excellent safety profile [32]. The broad therapeutic potentials of losartan have also been showcased in fibrotic disorders in the heart, lung, liver, and kidney [33–36]. Although information about the anti-fibrotic effects of losartan in patients with HPS-1 pulmonary fibrosis and hypertension is not available, losartan stabilized lung function in patients with idiopathic pulmonary fibrosis and had antifibrotic effects in the livers of patients with nonalcoholic steatohepatitis and hypertension [36, 37]. Collectively, these properties render losartan a conceptually attractive candidate for HPS pulmonary fibrosis treatment or perhaps prophylaxis against the development of pulmonary fibrosis.

Manifestations associated with BLOC-3 defects are extensively studied in cells possessing LRO [16, 17, 38]. Here, we demonstrate that normal human lung fibroblasts express functional BLOC-3 despite the lack of LRO. It is also noteworthy that the BLOC-3 defect in HPSLF upregulated *MYH10* expression, which encodes Myosin IIB. Knockdown of *HPS1* or *HPS4* in NLF recapitulates the increase in Myosin IIB protein featured by HPSLF, thus supporting our findings that intrinsic BLOC-3 defects contribute to Myosin IIB levels accumulation. However, given that the increase in Myosin IIB is driven by increased RNA levels and not trafficking defects, further studies are warranted to elucidate mechanisms of aberrant MYOSIN IIB accumulation in HPS-1.

The underlying causes of HPS pulmonary fibrosis are most likely multifaceted. Dysfunction of alveolar epithelial cells and macrophages has emerged as a critical contributor to disease pathogenesis [39], although the role of lung fibroblasts is largely unknown. Intriguingly, our in vitro wound healing assays reveal the increased migration velocity of HPSLF and *HPS1*- silenced NLF. In light of the deleterious impact of unchecked fibroblast migration on tissue remodeling [40], our observations strongly support the prominent role of HPSLF in promoting lung fibrogenesis. Mechanistically, we show that the kinase activity of p38 MAPK is required for the downstream upregulation of Myosin IIB in HPSLF. Furthermore, we observed that abrogation of Myosin IIB using genetic ablation of *MYH10* or pharmacological inhibition of AGTR1 with losartan mitigates the migratory phenotype of HPSLF in vitro and potentially in vivo, thus highlighting the role of the AGTR1-p38 MAPK-Myosin IIB pathway for fibroblast motility. Since HPS is rare disease, the number of primary fibroblasts used in these experiments is limited, which is a limitation of our work. However, we believe that our data are strengthened by the genetic manipulation of HPS1, HPS4 and Rab32 in normal lung fibroblasts.

In summary, our findings unravel a previously unrecognized role of BLOC-3 in human lung fibroblasts and offer novel molecular insights into HPSLF migration. Our study supports a role for aberrant Myosin IIB in HPS pulmonary fibrosis and provides an in vitro validation of losartan as a viable potential therapy. Future studies are needed to better understand the mechanisms of Myosin IIB accumulation and to evaluate the efficacy of AGTR1 inhibitors in animal models of HPS pulmonary fibrosis.

## Methods

### Human lung tissues collection

Under protocol 04-HG-0211 approved by the Institutional Review Board (IRB) of the National Human Genome Research Institute, lung tissues from healthy research volunteers and subjects with HPS-1 pulmonary fibrosis undergoing lung transplantation were procured as described previously [20].

Cell Culture**.** Primary human lung fibroblasts were obtained by explant cultures from control healthy lungs (NLF) and HPS-1 fibrotic lungs (HPSLF) as described previously [20]. HPS lung fibroblasts were cultured from lung explants donated by 1 female and 1 male; one patient was in their late 40’s and one patient was in their early 50’s. One normal lung fibroblast donor was a 63-year-old female, and another donor was a 67 year old male. The Cells were maintained in Dulbecco's Modified Eagle Medium Media (Thermo Fisher Scientific, Rockford, IL) with 10% FBS and incubated at 37 °C in a humidified 5% CO_2_ atmosphere. The cells were passaged every 3 days with an average doubling time of 33 h and could be maintained until passage 10–11. For all included experiments the cells were used between passages 5–9. Cells were serum-starved overnight before they were treated with losartan (100 nM) (TOCRIS, Minneapolis, MN) or SB202190 (5 µM) (Cayman Chemicals, Ann Arbor, MI) for the indicated times.

### RNA interference

The siRNAs for human *HPS1*, *HPS4*, *MYH10*, and *RAB32* were designed with the Whitehead siRNA Selection Web Server as described previously [41] and were synthesized by Sigma-Aldrich. Refer to (Additional file 5: Table S1) for siRNA sequences. Cells were transfected with either non-targeting control siRNA (MISSION siRNA Universal Negative Control, Sigma-Aldrich) or specific siRNA to suppress the expression of the intended gene. Transfection was carried out with 10 nmol/L siRNA by using Lipofectamine RNAiMAX (Thermo Fisher Scientific, Rockford, IL), according to the manufacturer’s recommendations. The efficiency of siRNA-mediated knockdown was confirmed by Western blotting.

### Flow cytometry

The fluorescence signal of CFSE labeled cells were measured using a FACSCanto II flow cytometer (BD Biosciences, Franklin Lakes, NJ). Data analysis was performed by using FlowJo (TreeStar, Ashland, OR).

### Western blotting

Cells were lysed with radioimmunoprecipitation assay (RIPA) buffer supplemented with protease and phosphatase inhibitors (Thermo Fisher Scientific). Protein concentration was subsequently determined with a BCA protein assay (Thermo Fisher Scientific). Equal amounts of protein extract from each sample were resolved by sodium dodecyl sulfate–polyacrylamide gel electrophoresis (SDS-PAGE) and transferred onto polyvinylidene fluoride (PVDF) membranes. The membrane was blocked with 5% skim milk in Tris-buffered saline with Tween (TBST) for 1 h at room temperature and then probed with primary antibody overnight at 4 °C. A complete list of antibodies is included in (Additional file 5: Table S2). After TBST wash, the membrane was incubated with the appropriate horseradish peroxidase (HRP)-conjugated secondary antibody (Thermo Fisher Scientific) for 1 h at room temperature. Blots were developed with SuperSignal West Pico chemiluminescent substrate (Thermo Fisher Scientific) and visualized by ChemiDoc XRS + imaging system (Bio-Rad). Imagelab (Bio-rad Laboratories, Hercules, CA).

### Scratch assay

Scratch assay was performed as previously described [22]. Briefly, cells were cultured to confluence in 60 mm and starved overnight. At time 0, the cell monolayer was scratched with a 1000-μl pipette tip and washed with PBS to remove floating cells. Phase-contrast images were captured at specified time points after scratching and analyzed with ImageJ. The rate of cell migration was determined by calculating the percentage of residual wound area at the indicated time points to that of the corresponding original wound.

### Cell proliferation assay

Cell proliferation was determined by a fluorescence-based assay that quantifies CFSE dilution in proliferating cells. NLF and HPSLF cells were stained with 5uM CFSE (Biolegend), 50% of each cell suspension was then treated with 10ug/ml Mitomycin C. The cells were then cultured for 3 days at which cell point the CFSE fluorescence was measured on a FACSCanto II flow cytometer (BD Biosciences, Franklin Lakes, NJ). The cell growth rate was calculated as the ratio of the MFI of CFSE in the mitomycin C treated cells over the untreated cells for both NLF and HPSLF.

### RNA extraction and real-time polymerase chain reaction

Total RNA was isolated with RNeasy Mini Kit (Qiagen). 1 μg RNA was reverse-transcribed with amfiRivert cDNA Synthesis Master Mix (GenDEPOT, Barker, TX, USA). A total of 1 μL of the resultant cDNA was subjected to a quantitative real-time polymerase chain reaction (PCR) with the StepOnePlus Real-Time PCR System (Thermo Fisher Scientific) by using RT^2^ SYBR Green qPCR Master Mix (Qiagen). Primers were designed by Primer Premier 5 software (Premier Biosoft International, Palo Alto, CA) and synthesized by Sigma-Aldrich. Refer to Additional file 5: Table S3 for primer sequences. Thermal cycling conditions were 95 °C for 10 min followed by 40 cycles of 95 °C for 15 s and 60 °C for 1 min. Gene expression was normalized to housekeeping gene 18S rRNA (18S) and determined by using the 2^−ΔΔCt^ method. Each sample was analyzed in triplicate and melting curve analysis was carried out to confirm the specificity of the primers.

### Immunofluorescence microscopy

NLF and HPS cells were cultured in 4 well chamber slides, the slides were washed twice with cold PBS and then fixed with 4% paraformaldehyde. The cells were then permeabilized with 0.1% Triton-X100 in PBS. Cells were washed twice with PBS and then incubated with Rabbit anti-Myosin IIB antibody overnight. The slide was washed twice with PBS and incubated with AlexaFluor 647 anti-rabbit secondary antibody and AlexaFluor 488 conjugated Phalloidin (Thermofisher Cat# A12379) following the manufactures instructions. Stained slides were imaged using an Olympus FluoView FV-10i confocal laser-scanning microscope (Olympus, Tokyo, Japan).

### Injections and analysis of human fibroblast cells in zebrafish embryos

All zebrafish work was done in the Tg(fli1:GFP) line [42] using an Animal Study Protocol (G-05–5 to RS) approved by the National Human Genome Research Institute’s Animal Care and Use Committee. Embryos were generated from a breeding stock, maintained at 28 °C in E3 water, and treated with 0.3 mg/mL PTU (N-Phenylthiourea) from 24 h post-fertilization (hpf) to suppress pigmentation. At 48 hpf, embryos were manually dechorionated, and anesthetized with 0.16 mg/ml tricaine (Syndel, Ferndale, WA). Seventy-five freshly thawed passage 4 HPSLFs or NLFs were suspended in 4 nL of 3 mg/mL Matrigel (Corning, Corning, NY) and were injected into the yolk sac of 48 h post-fertilization zebrafish embryos using a microinjector (World Precision Instruments, Sarasota, FL). Zebrafish embryos were screened for CM-Dil signal using a Leica MZ16F microscope (Leica, Wetzlar, Germany) 2 h after injection. Tile images from zebrafish mounted on glass-bottom microwell dishes (MatTek, Ashland, MA) in 0.8% low-melt agarose (Sigma-Aldrich, St. Louis, MO) containing 0.16 mg/mL tricaine were obtained at 24 and 48 h after injection using a Zeiss LSM 810 confocal microscope (Zeiss, Oberkochen, Germany). At least 20 zebrafish embryos were analyzed for experiments performed in triplicate using cells from 2 different patients with HPS-1 pulmonary fibrosis.

### Statistical analysis

Data are presented as mean ± standard error of the mean (SEM) from three independent experiments. Comparisons between two groups were performed with a two-tailed Student *t*-test. Variables with more than two factors were evaluated by one-way analysis of variance (ANOVA) followed by the Tukey post hoc test. Scratch Assay results were analyzed using two-way ANOVA followed by the Sidak post hoc test. Statistical analyses were carried out with GraphPad Prism 9.0.2 (GraphPad Software, La Jolla, CA). *P* < 0.05 was considered significant.

## Supplementary Information

**Additional file 1: Fig S1.** Real-time PCR analysis of **A** *COL1A1*, **B** *COL3A1*, **C** *COL4A1*, **D** *ELN*, **E** *LAMB1*, **F** *FLN*, **G** *TIMP3*, **H** *HAS1*, **I** *HAS2*, **J** *HAS3* mRNA in NLF (n = 3 technical replicates) and HPSLF (n = 3 technical replicates). Results were expressed as fold change relative to NLF. Data are expressed as mean ± SEM of three independent experiments. Data analyzed using a student’s *t*-test *P < 0.05, **P < 0.01, ****P < 0.0001

**Additional file 2: Fig S2.** **A** Western blot analysis of Myosin IIB and HPS1 Protein expression from independent NLF and HPSLF patient cells are shown; β-Actin was used as a loading control. **B** Ratio of Myosin IIB to β-Actin density expressed as fold-change relative to control NLF. **C** Western blot analysis of Myosin IIB and HPS1 Protein in NLF cells silenced with a second HPS1 siRNA construct; β-Actin was used as a loading control. **D** Ratio of Myosin IIB to β-Actin density expressed as fold-change relative to control siRNA treated cells. Data are expressed as mean ± SEM of three independent experiments. Data analyzed using a student’s *t*-test *P < 0.05, **P < 0.01

**Additional file 3: Fig S3.** **A** Western blot analysis of Myosin IIB in HPSLF (n = 3 technical replicates) treated with vehicle (DMSO) or with SB202190 (5 µM). β-Actin was used as a loading control. **B** Ratio of Myosin IIB to β-Actin density expressed as fold-change relative to DMSO. **C** Western blot analysis of phospho-p38 and p38 in NLF (n = 3 technical replicates) transfected with control or 2 different HPS1 siRNA constructs. β-Actin was used as a loading control. **D** Ratio of phospho p38 to p38 Density; the ratio of each HPS1 transfected NLF was compared to its own control siRNA transfected cell. **E** Western blot analysis of phospho-p38 and p38 in NLF (n = 3 technical replicates) transfect with control or HPS4 siRNA. β-Actin was used as a loading control. (*F*) Ratio of phospho p38 to p38 Density. Data are expressed as mean ± SEM of three independent experiments. Data analyzed using a student’s t-test *P < 0.05, **P < 0.01, ***P < 0.001, ****P < 0.0001

**Additional file 4: Fig. S4.** Losartan Modulates the migratory capacity of HPSLF in vivo. NLF (Right) and HPSLF (Left) were labeled with CM-Dil live-cell marker (red) and seventy-five cells were injected into the yolk sac of 48 h post-fertilization Tg(fli1:GFP, green) zebrafish embryos expressing GFP in their blood vessels (n = 5 biological replicates). At 48 post-injection, HPSLF migrated farther than NLF (column 3 vs 1). Treatment of HPSLF with losartan reduced the migratory capacity compared to control treatment (Column 3 vs Column 4).

**Additional file 5:** **Table S1.** siRNA sequences. **Table S2.** Complete list of antibodies. **Table S3.** Real-time PCR primer sequences.

## Data Availability

All data generated and analyzed during this study are included in this published article (and its additional files).
