# Supplementary figures and images for: Dysregulated myosin in Hermansky-Pudlak syndrome lung fibroblasts is associated with increased cell motility
Source: Respir Res. 2022 Jun 23;23:167. doi: 10.1186/s12931-022-02083-w (PMC9229912; doi:10.1186/s12931-022-02083-w)

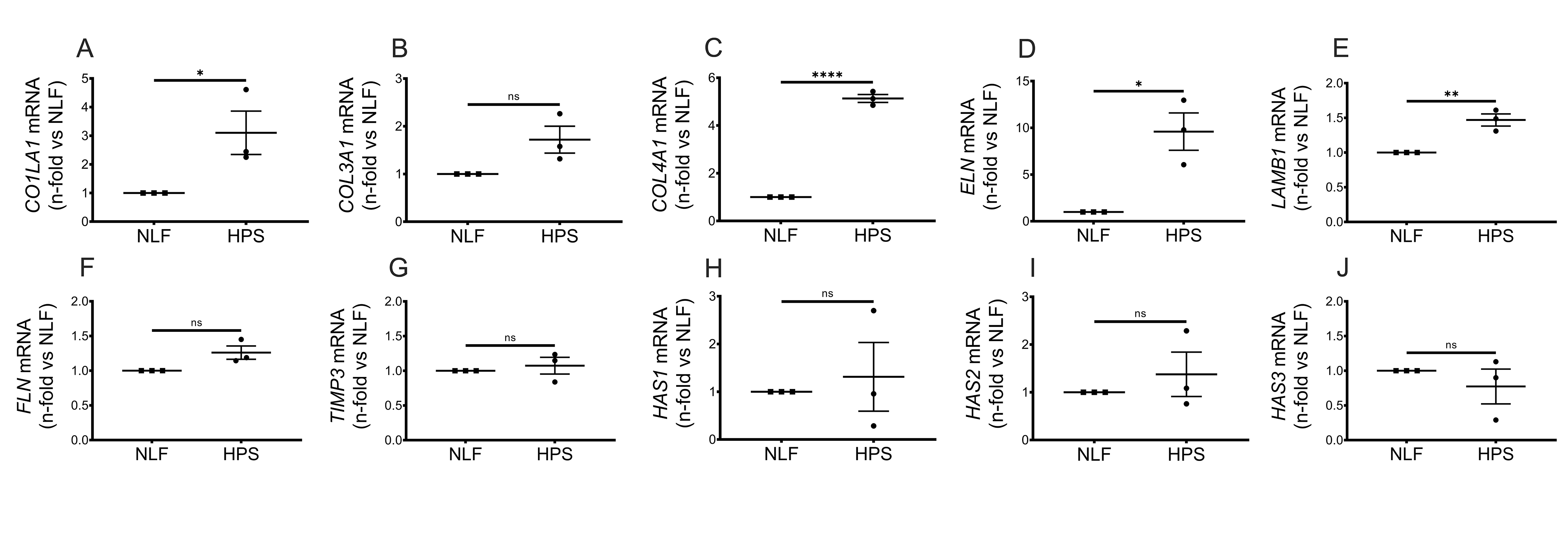

Supplement: Supplementary file 1 — Additional file 1: Fig S1. Real-time PCR analysis of A COL1A1, B COL3A1, C COL4A1, D ELN, E LAMB1, F FLN, G TIMP3, H HAS1, I HAS2, J HAS3 mRNA in NLF (n = 3 technical replicates) and HPSLF (n = 3 technical replicates). Results were expressed as fold change relative to NLF. Data are expressed as mean ± SEM of three independent experiments. Data analyzed using a student’s t-test *P < 0.05, **P < 0.01, ****P < 0.0001 [file 12931_2022_2083_MOESM1_ESM.tif]

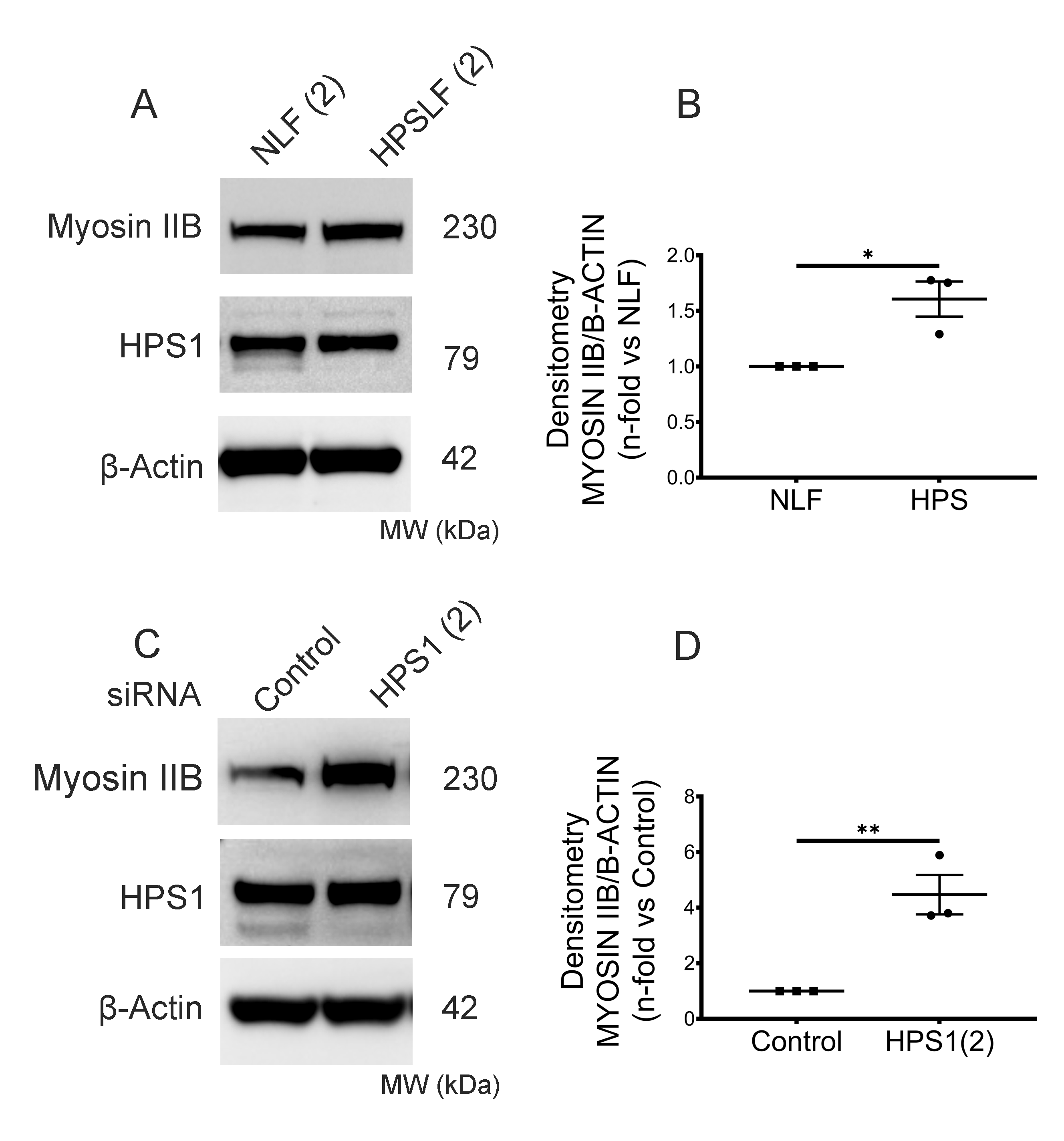

Supplement: Supplementary file 2 — Additional file 2: Fig S2. A Western blot analysis of Myosin IIB and HPS1 Protein expression from independent NLF and HPSLF patient cells are shown; β-Actin was used as a loading control. B Ratio of Myosin IIB to β-Actin density expressed as fold-change relative to control NLF. C Western blot analysis of Myosin IIB and HPS1 Protein in NLF cells silenced with a second HPS1 siRNA construct; β-Actin was used as a loading control. D Ratio of Myosin IIB to β-Actin density expressed as fold-change relative to control siRNA treated cells. Data are expressed as mean ± SEM of three independent experiments. Data analyzed using a student’s t-test *P < 0.05, **P < 0.01 [file 12931_2022_2083_MOESM2_ESM.tif]

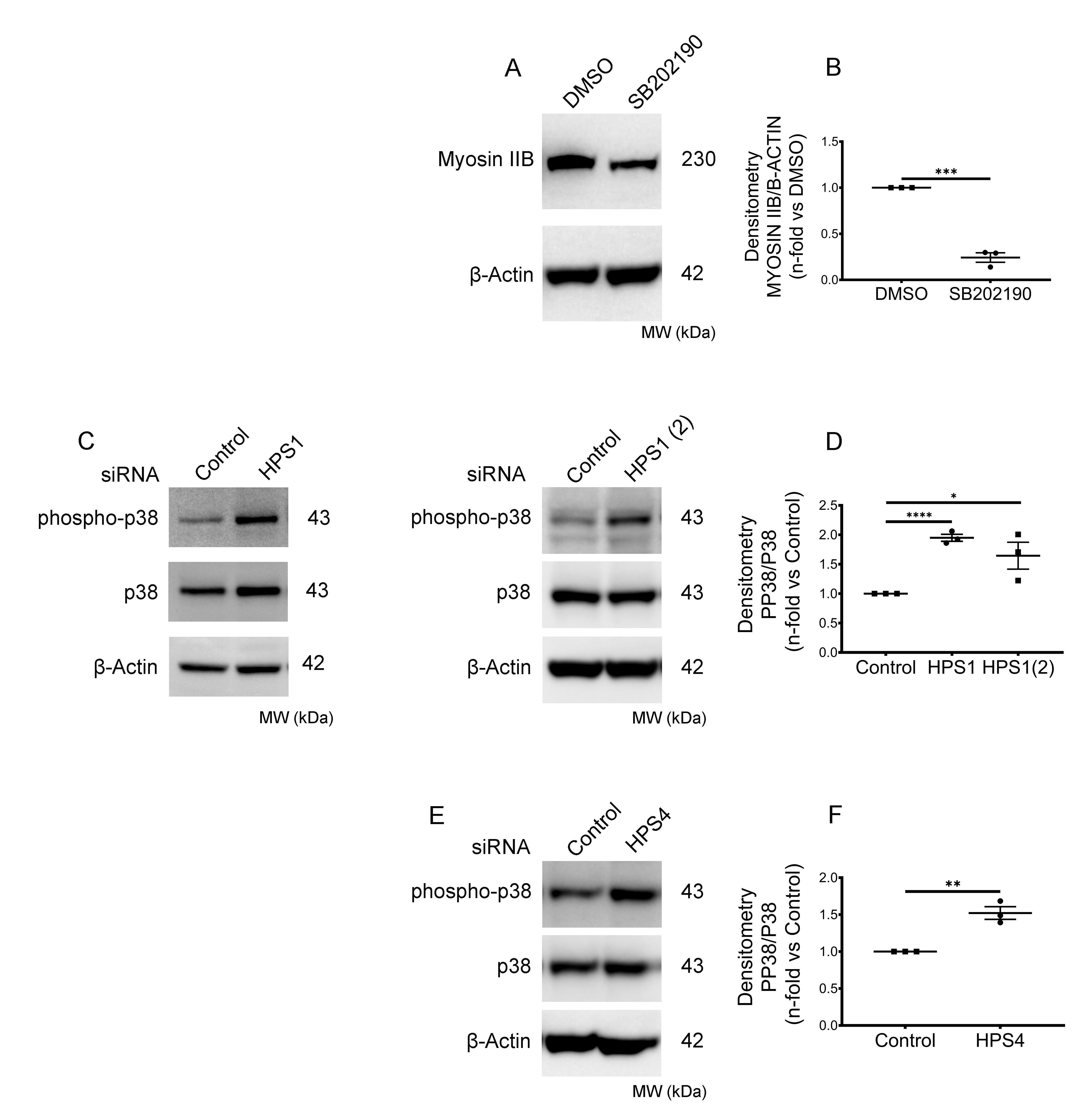

Supplement: Supplementary file 3 — Additional file 3: Fig S3. A Western blot analysis of Myosin IIB in HPSLF (n = 3 technical replicates) treated with vehicle (DMSO) or with SB202190 (5 µM). β-Actin was used as a loading control. B Ratio of Myosin IIB to β-Actin density expressed as fold-change relative to DMSO. C Western blot analysis of phospho-p38 and p38 in NLF (n = 3 technical replicates) transfected with control or 2 different HPS1 siRNA constructs. β-Actin was used as a loading control. D Ratio of phospho p38 to p38 Density; the ratio of each HPS1 transfected NLF was compared to its own control siRNA transfected cell. E Western blot analysis of phospho-p38 and p38 in NLF (n = 3 technical replicates) transfect with control or HPS4 siRNA. β-Actin was used as a loading control. (F) Ratio of phospho p38 to p38 Density. Data are expressed as mean ± SEM of three independent experiments. Data analyzed using a student’s t-test *P < 0.05, **P < 0.01, ***P < 0.001, ****P < 0.0001 [file 12931_2022_2083_MOESM3_ESM.tif]

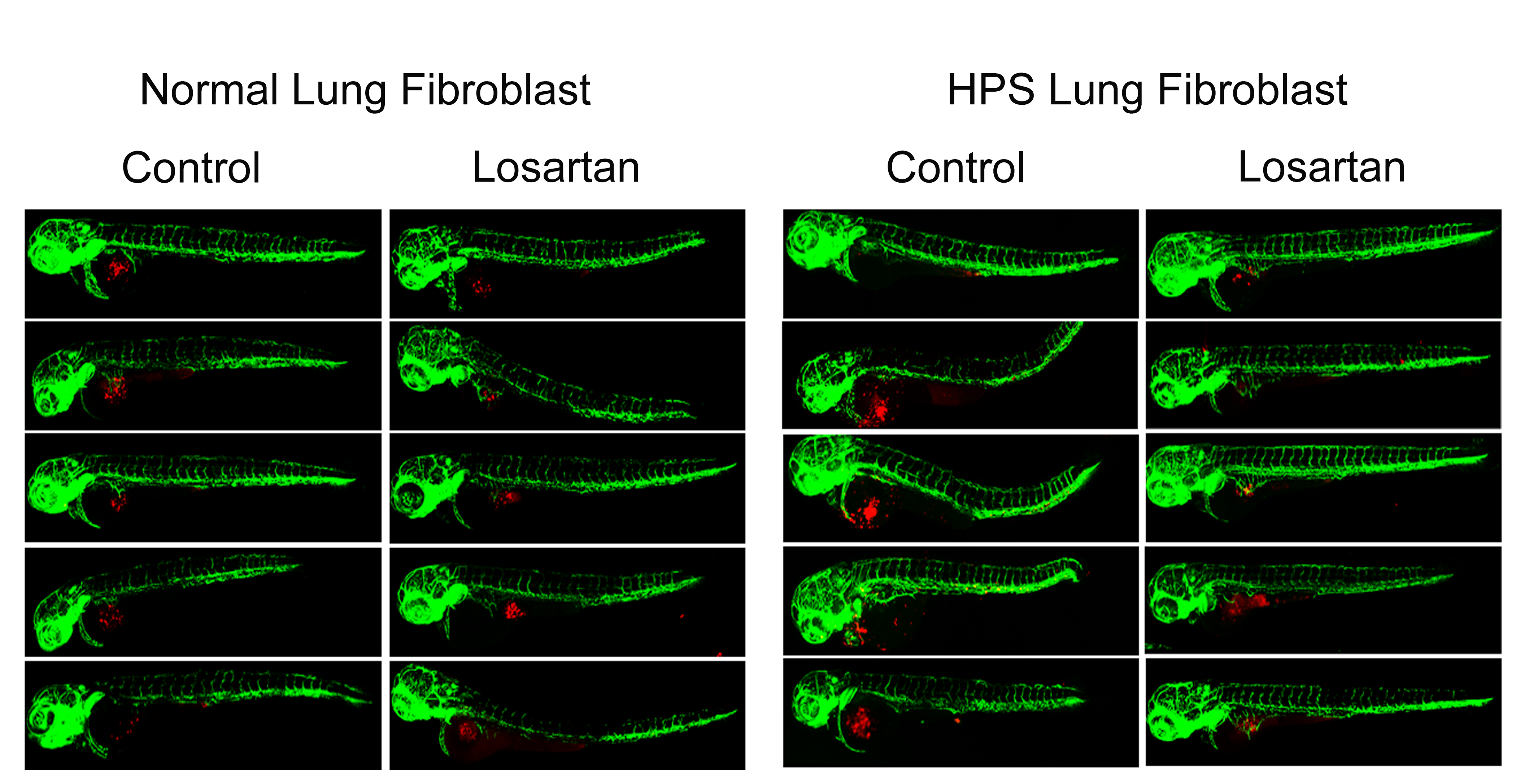

Supplement: Supplementary file 4 — Additional file 4: Fig. S4. Losartan Modulates the migratory capacity of HPSLF in vivo. NLF (Right) and HPSLF (Left) were labeled with CM-Dil live-cell marker (red) and seventy-five cells were injected into the yolk sac of 48 h post-fertilization Tg(fli1:GFP, green) zebrafish embryos expressing GFP in their blood vessels (n = 5 biological replicates). At 48 post-injection, HPSLF migrated farther than NLF (column 3 vs 1). Treatment of HPSLF with losartan reduced the migratory capacity compared to control treatment (Column 3 vs Column 4). [file 12931_2022_2083_MOESM4_ESM.tif]
