# Supplementary material for: Dysregulated myosin in Hermansky-Pudlak syndrome lung fibroblasts is associated with increased cell motility
Source: Respir Res. 2022 Jun 23;23:167. doi: 10.1186/s12931-022-02083-w (PMC9229912; doi:10.1186/s12931-022-02083-w)
Supplement: Supplementary file 5 — Additional file 5: Table S1. siRNA sequences. Table S2. Complete list of antibodies. Table S3. Real-time PCR primer sequences. [file 12931_2022_2083_MOESM5_ESM.docx]

**Additional file 5: Tables**

**Table S1. siRNA sequences.**

| **Target gene/**  **accession number** | **Sequence 5′ to 3′** | |
| --- | --- | --- |
| *HPS1*/ NM_000195 | Sense | GUCAUCAUCUCCUCCAUGA[dT][dT] |
|  | Anti-sense | UCAUGGAGGAGAUGAUGAC[dT][dT] |
| *HPS1(2)*/NM_000195 | Sense | CAUGGUGUCCUACCUAGAA[dT][dT] |
|  | Anti-sense | UUCUAGGUAGGACACCAUG[dT][dT] |
| *HPS4/* NM_022081 | Sense | GGAUGAAUCUCUACACUCA[dT][dT] |
|  | Anti-sense | UGAGUGUAGAGAUUCAUCC[dT][dT] |
| *MYH10*/ NM_005964 | Sense | GCAAUACAGUGGGACAGUU[dT][dT] |
|  | Anti-sense | AACUGUCCCACUGUAUUGC[dT][dT] |
| *RAB32*/  NM_006834 | Sense | GGGUUACAGAUGUCAUGUU[dT][dT] |
|  | Anti-sense | AACAUGACAUCUGUAACCC[dT][dT] |

**Table S2. Complete list of antibodies.**

| **Antigen** | **Application** | **Source** |
| --- | --- | --- |
| β-Actin | Immunoblotting | Catalog # A2228 (Sigma-Aldrich, St. Louis, MO) |
| HPS1 | Immunoblotting | Catalog # ab174301 (Abcam, Cambridge, MA) |
| HPS4 | Immunoblotting | Catalog # 14627-1-AP (Rosemont, IL) |
| MYOSIN IIB | Immunoblotting Immunofluorescence | Catalog # 8824 (Cell Signaling Technology, Danvers, MA) |
| phospho-p38 MAPK | Immunoblotting | Catalog # 4511 (Cell Signaling Technology) |
| p38 MAPK | Immunoblotting | Catalog # 8690 (Cell Signaling Technology) |
| Rab32 | Immunoblotting | Catalog # SAB4200086 (Sigma-Aldrich) |

**Table S3. Real-time PCR primer sequences.**

| **Target gene/ accession number** | **Sequence 5′ to 3′** | |
| --- | --- | --- |
| *18S*/ NM_004048 | Sense | ATGGCCGTTCTTAGTTGGTG |
|  | Anti-sense | CGCTGAGCCAGTCAGTGTAG |
| *LAMB1/* NM_002291 | Sense | GTGTGTATAGATACTTCGCC |
|  | Anti-sense | AAAGCACGAAATATCACCTC |
| *COL1A1/*NM_000088 | Sense | GCTATGATGAGAAATCAACCG |
|  | Anti-sense | TCATCTCCATTCTTTCCAGG |
| *COL3A1/* NM_000090 | Sense | ATTCACCTACACAGTTCTGG |
|  | Anti-sense | TGCGTGTTCGATATTCAAAG |
| *COL4A1/* NM_001845 | Sense | AAAGGGAGATCAAGGGATAG |
|  | Anti-sense | TCACCTTTTTCTCCAGGTAG |
| *ELN*/ NM_000501 | Sense | TAAAGCAGCTAAATACGGTG |
|  | Anti-sense | AGGAAGCTCATTTTCTCTTC |
| *FN1*/ NM_002026 | Sense | CCATAGCTGAGAAGTGTTTG |
|  | Anti-sense | CAAGTACAATCTACCATCATCC |
| *TIMP3*/ NM_000362 | Sense | CATGTGCAGTACATCCATAC |
|  | Anti-sense | AGGTGATACCGATAGTTCAG |

| *MYH10*/ NM_005964 | Sense | CGGTGGGTTTGGGACTGAG |
| --- | --- | --- |
|  | Anti-sense | CTCGAGTCCAGTTCTCTGCG |
